# Treatment of War Pensioners and Other

**Published:** 1917-11-17

**Authors:** 


### PAYMENTS FOR PATIENTS IN VOLUNTARY HOSPITAL

### TREATMENT OF WAR PENSIONERS AND [OTHERS.

A PAMPHLET of instructions recently issued by the Ministry of Pensions as to the treatment of war pensioners has stirred the managers of voluntary hospitals to consider their position. In Schedule 1, on page 17 of these instructions, the Minister assumes that the voluntary hospitals will undertake the treatment of these patients, and this without any previous consultation with the hospital or negotiation as to the terms on which such assistance should be given. We shall no doubt be perfectly right in concluding that all voluntary hospitals will gladly recognise their duty to render every possible assistance in their power to those wounded heroes who have done so much for their country, and will undoubtedly place their resources at the country's disposal to ameliorate the condition of these men. But surely this is a matter on which it was the duty of the Ministry to consult the hospital managers. It is our pride that our great hospitals have risen to their prominent position as a result of their voluntary character. Founded in the first instance by a few generous philanthropists, these hospitals have won the support of the public, they have been supported by the philanthropic rich and they have been resorted to in huge numbers by those who need and those who deserve their help. Almost invariably they have been wisely administered and all have prospered and grown in strength and usefulness. They could not have won the support of the rich without deserving this by wise administration, they would not have served the purpose for which they were founded and secured the confidence of those who need to resort to them in times of sickness and accident had they not always been ready to render efficient and satisfactory—and may we add, judicious?—help to the needy. It is to them that we owe the great progress which has been made in the modern developments of medicine and surgery, and it may safely be said that they have wisely done their duty.

The English nation has never taken kindly to bureaucracy in any form. State hospitals of various kinds, whether administered by branches of the Services or by Municipalities, have never attained to the high standard of work which has been the hallmark of these voluntary hospitals, and it will assuredly be wise that if possible this admirable system be maintained; but the time has now arrived when the question must be resolutely faced and a wise solution evolved.

### GRANTS IN AID.

What alterations are necessary in the management of these hospitals to meet the needs of the present, and to define a wise policy for the future? This decision of the Ministry of Pensions brings the matter home to all thinking men. For some years past many of these hospitals, which for years had depended entirely on voluntary contributions, have been receiving payments which may be considered as nothing more than grants in aid. The most notable of these, perhaps the turning-point in the change of system, came with the passing of the Insurance Act in 1911, which led to the establishment of tuberculosis dispensaries and the definite payment of a sum of money in recognition of the treatment of these cases in those dispensaries. Now it was necessary for the hospitals not to shrink from undertaking this work. Those hospitals which had medical schools attached to them would have lost much of the opportunity of training their men had they lost all tuberculosis patients. This system of payment having been recognised, it was not long before the Chief Commissioner of Metropolitan Police made an arrangement for the payment of what is still called a subscription to hospitals in recognition of the treatment within their walls of police cases who might be referred to them by the responsible medical officers. These policemen are of the class of person who would in ordinary life be regarded as fit and proper persons for treatment in voluntary hospitals as cases deserving the charity of those institutions. How far the police authorities who are responsible for the treatment of their men while sick were justified in taking the assistance of the voluntary hospitals for a payment of £1 1s. per week for in-patients, when the average cost of an in-patient in most of the Metropolitan hospitals is at least twice as much, is a matter into which we need not go now, though, incidentally, it may be mentioned that no payment is made for their treatment as out-patients.

### THE COUNTY COUNCIL'S WISE ACTION.

Then came the question of the treatment of venereal disease, which was handed over to, so far as the Metropolis is concerned, the control of the London County Council, and they, wisely we think, made a selection of twenty-two hospitals which they thought should be invited to assist in the scheme, and to which they undertook to make a definite monetary payment. It is rather early yet to judge the success of this scheme, but it promises well. It was tentative in the first instance, and no doubt injustice will be adjusted and any glaring faults rectified in the scheme as the result of this first year's working; but on the whole we think that the matter was wisely approached, the hospitals were individually consulted, and on their estimate of the number of patients treated and their capacity to make arrangements to deal with these cases, a definite sum was agreed as the payment for the first year to cover the cost both of in-patients and of out-patients. The most notable point of all in this scheme is that adequate payment is made to the

**PAYMENTS FOR PATIENTS IN VOLUNTARY HOSPITALS—(*continued*).**

medical men engaged in treating the patients. An annual sum was paid to the governors of the hospital, and it rested with them, as a matter of internal administration, to decide what proportion of it would be an adequate remuneration of their staff engaged in this special work. Now in both these instances, of the tuberculosis dispensary attached to a big voluntary hospital and the treatment of venereal disease in a special department of the hospital, definite diseases are dealt with, but in the problem before us the Ministry have to deal with every imaginable disease that flesh is heir to, and to provide efficient post-treatment for every kind of injury. This will need the full equipment of our best hospitals, and will require further the services of each and all of the voluntary staff attached thereto.

### Pensions Committee's Scale is Inadequate.

As the Ministry has undertaken the treatment of these pensioners, it is no doubt prepared to pay the full cost of this treatment. We are the more surprised, therefore, at the issue of the scale of payment which the Local War Pensions Committees are authorised to make to managers of institutions:—

28s. per week for in-patients.

1s. for first attendance and 6d. for each subsequent attendance for out-patients, or

2s. 6d. for first attendance and 1s. for subsequent attendance in special departments.

An examination either of the reports of the various hospitals or of the tables which are issued either in "Burdett's Hospitals and Charities" or in the returns of King Edward's Hospital Fund, at once shows that this is a scale of payment very considerably below the actual cost of each patient's treatment either as an in-patient or as an out-patient. The Local War Pensions Committee's scale will be recognised by all who know anything of hospital work as totally inadequate to reimburse the managers the actual outlay involved. There may or may not be any considerable number of these people; but, whatever the number, is the Ministry justified in assuming that the voluntary staffs of these hospitals should be asked to give their valuable time in the treatment of cases for which the State has made itself responsible?

### The Position of Medical Staffs.

The visiting staffs of our hospitals are honorary. We need not consider for the moment why they are prepared to undertake the work of attending on the sick poor in these great institutions without any fixed payment, but we may say that they have been, at least up to the present, so far content with the return for the services they render that they have been, and we believe are, satisfied to continue to work on these terms. They are not content to undertake this new work for the State without adequate payment, nor should they be asked to do so.

We thus come to the crux of our problem. Can the work in the hospitals be so organised as to undertake the treatment of war pensioners and to provide an adequate remuneration for the medical staff without destroying the voluntary principle under which the staff at present work? In the two cases which we have instanced, the treatment of tuberculosis patients for the Insurance Committee and the treatment of venereal disease for the London County Council, medical officers have been appointed by the governors at a salary paid by the governors for their work, and the honorary staff each in their special departments can, and we believe are prepared to, continue to give the benefit of their supervision as consulting referees in all difficult or interesting cases.

### Can the Voluntary System and Work that is Paid for be Efficiently Carried on Side by Side?

In one large hospital, at least, we have a Paying Home attached. To this patients are admitted who are prepared to make an adequate payment for their maintenance and treatment in the Home, and in addition pay the fees of the consultants whom they employ. In so liberal a spirit is this Home administered that the patients are not restricted to the staff of that hospital in making a selection, but may employ members of the honorary staff of any recognised general hospital.

### A Successful Partial System of Payments.

At the same hospital, which is administered under its Charter and various Acts of Parliament, power rests with the governors to make a charge for in-patient treatment to any individuals who are admitted, and whose social circumstances warrant such charge being made. To carry out this system efficiently a large department strictly confining itself to social work amongst patients has grown up, and by the efficient aid of the lady almoners in charge the charitable funds of the hospital are protected from imposition. Cases which without making a payment can hardly be called proper cases for admission are admitted for treatment in the wards. £1 1s. per week is the maximum payment which may be taken from these patients. If the patients can pay more, then they are suitable for admission to the Home, to which they are referred.

### Full and Adequate Payment.

In the Home Hospital the staff receive full and adequate payment, but for the cases in the general hospital they neither receive nor expect any payment.

### Facts for Hospital Managers.

This necessarily short summary, inadequate possibly in many of its particulars, is devised to lay the facts before the Hospital Managers. If they give their minds to it there is no doubt that they will themselves come to a satisfactory decision. By uniting their efforts they can put a strong case before the Ministry of Pensions, and we have every hope of their attaining a successful solution of the problem.

### How the Hospitals can Aid the Government.

In what way can the voluntary hospitals most usefully aid the Government's great scheme? Primarily the Hospitals should form consulting and

**PAYMENTS FOR PATIENTS IN VOLUNTARY HOSPITALS—** *(continued).*

advising centres. It should not be contemplated that they will undertake the treatment of ordinary medical and surgical cases which it is well within the power of general practitioners and panel doctors to deal with. To them all such cases should be referred. The hospitals, if their work is properly organised, should form a valuable protection and shield to the Ministry against imposition and fraud. The best men alone can say that a patient needs no treatment, and their decisions should carry such weight as to be final. It is suggested that as a practical method of dealing with this matter each hospital should form from the members of its staff a small examining board, to whom all cases would be referred on coming to the hospital for treatment. It would be the duty of this board to assign these cases, some to the general practitioners, the rest to the various departments of the hospital, where they can receive the special treatment they need. We have only to instance cases needing *x*-ray treatment, massage, ophthalmic, aural, and other treatment to realise the necessity of the hospitals undertaking quite a large proportion of the work.

### A Convenient Scheme of Payment.

A convenient method of payment of the staff for the valuable services they are asked to undertake as members of the examining board would be to pay them on a similar scale to that on which medical officers of insurance companies are paid—so much per meeting—and this payment the Ministry of Pensions would be wise to make itself responsible for, in the shape of a quarterly payment to the managers of the hospital for allocation to the members of the staff who do the work. The payment for the maintenance and treatment given in the hospital both for in-patients and out-patients should be adequate; the figures are available, and the actual cost per in-patient and per out-patient attendance should be paid.

### A Final Point of Importance.

There then remains but one further point to be dealt with. All these cases are docketed, and to each man's dossier must be attached reports on his case. These reports will be of the utmost importance to the individual pensioners, for on them will largely depend the continuance or the fluctuation in the value of the pension. An agreed reasonable fee for each report of a full and technical nature should be paid to the managers of the hospital, to be distributed by them to the individual members of the staff who make the report.

It is suggested that under this system the honorary character of the work of the consultants, as the staff of the various voluntary hospitals, will be maintained. Further, the individual consultants, as professional men, will receive adequate payment for the work they undertake to do, for which the Government holds itself responsible.

